# Evidence for a cordal, not ganglionic, pattern of cephalopod brain neurogenesis

**DOI:** 10.1186/s40851-015-0026-z

**Published:** 2015-09-07

**Authors:** Shuichi Shigeno, Rahul Parnaik, Caroline B. Albertin, Clifton W. Ragsdale

**Affiliations:** Department of Marine Biodiversity Research, Japan Agency for Marine-Earth Science and Technology, Yokosuka, 237-0061 Japan; Department of Neurobiology, The University of Chicago, 947 E 58th Street, Chicago, IL 60637 USA; Department of Organismal Biology and Anatomy, The University of Chicago, 1027 E 57th Street, Chicago, IL 60637 USA

## Abstract

**Introduction:**

From the large-brained cephalopods to the acephalic bivalves, molluscs show a vast range of nervous system centralization patterns. Despite this diversity, molluscan nervous systems, broadly considered, are organized either as medullary cords, as seen in chitons, or as ganglia, which are typical of gastropods and bivalves. The cephalopod brain is exceptional not just in terms of its size; its relationship to a molluscan cordal or ganglionic plan has not been resolved from the study of its compacted adult structure. One approach to clarifying this puzzle is to investigate the patterns of early cephalopod brain neurogenesis, where molecular markers for cephalopod neural development may be informative.

**Results:**

We report here on early brain pattern formation in the California two-spot octopus, *Octopus bimaculoides*. Employing gene expression analysis with the pan-bilaterian neuronal marker *ELAV* and the atonal-related neuronal differentiation genes *NEUROGENIN* and *NEUROD*, as well as immunostaining using a Distalless-like homeoprotein antibody, we found that the octopus central brain forms from concentric cords rather than bilaterally distributed pairs of ganglia.

**Conclusion:**

We conclude that the cephalopod brain, despite its great size and elaborate specializations, retains in its development the hypothesized ancestral molluscan nervous system plan of medullary cords, as described for chitons and other aculiferan molluscs.

## Introduction

Molluscs are highly successful animals with a great diversity of body plans. Exceptional even among this group are the cephalopods, with the elaborate sensory systems, flexible learning abilities, and sophisticated motor outputs expected of an accomplished predator (reviewed in [1–3]). The behavioral capacities of cephalopods are reflected in the size and complexity of the cephalopod brain, with its dozens of functionally distinct lobes and associated complex of neural interconnections [4]. How cephalopod brain organization corresponds with patterns of neural tissue organization in other molluscs remains uncertain.

**Fig. 1:**
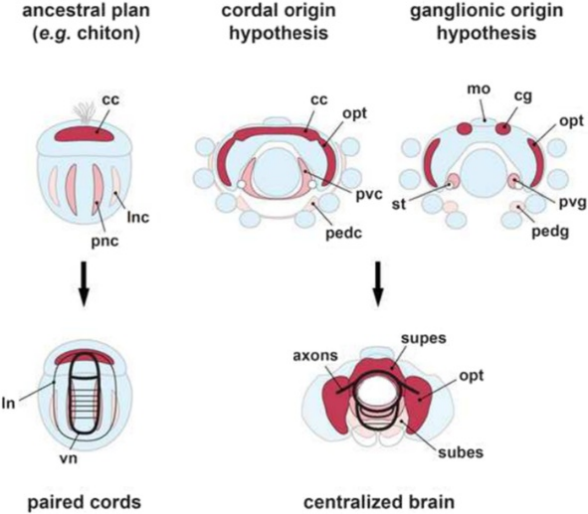
Traditional and alternative models for early brain development in cephalopods compared with the proposed ancestral cord-like nervous system of chitons. *Left Panels*, the cordal organization of the chiton nervous system. *Middle and Right Panels*, the competing hypotheses for a cordal (*Middle*) or ganglionic (*Right*) spatial pattern of octopus nervous system development. *Upper Panels*, cartoons of the chiton trochophore larva and coleoid cephalopod embryo with the apical organ, or mouth (mo), to the top. *Lower Panels*, cartoon of late embryogenesis when, in chitons (*Left)*, the major commissures and connectives are present and, in cephalopods (*Right*), the neuronal primordia have formed masses surrounding the esophagus. *Upper Right Panel*: Traditional section reconstruction studies concluded that brain neurons are initially clustered in spherical structures as ganglia [13, 14]. *Upper Middle Panel*: An alternative model in which the allocation of brain neurons is to cord-like territories similar to the arrangement described for polyplacophoran nervous systems. There is not yet a study of chiton neurogenesis employing pan-neuronal gene markers; the pattern illustrated on the left is inferred from immunocytochemical study of early and late larval stages [54, 56]. cc, cerebral cord; cg, cerebral ganglion; ln, lateral neurite bundle; lnc, lateral nerve cord; opt, optic lobe; pedc, pedal cord; pedg, pedal ganglion; pnc, pedal nerve cord; pvc, palliovisceral cord; pvg, palliovisceral ganglion; st, statocyst; subes, subesophageal mass; supes, supraesophageal mass; vn, ventral neurite bundle

Molluscan nervous systems are primarily organized as either medullary cords or ganglia^1^. Aculiferan molluscs, such as the chiton, have a nervous system based on cords, which are longitudinal neuropils surrounded by a rind of neuronal cell bodies [5]. In contrast, three of the major radiations of conchiferan molluscs (scaphopods, gastropods, and bivalves) have nervous systems featuring ganglia, which are tightly packed, clearly demarcated clusters of neuronal cell bodies that enclose a centralized neuropil (Fig. 1). The molluscan correlates of the cephalopod circumesophageal brain, with its extensive innovations in its organizational pattern, remain, however, obscure.

One approach to studying cephalopod brain evolution has been to investigate nautiloid cephalopods, an ancient lineage that includes the only extant non-coleoid cephalopods [6]. Young [7] described the adult brain of *Nautilus* as consisting of cerebral, pedal, and visceral cords surrounding the esophagus and suggested a homology between these cords and those of a polyplacophoran nervous system, such as that of a chiton [8, 9]. A problem for this interpretation is that the lobes of the *Nautilus* brain, unlike those of coleoid brains, show a mixing of neuronal somata and processes that does not follow the typical invertebrate histology of somata situated peripherally around a central core of neuropil. It is possible that the cord-like appearance of the *Nautilus* brain is a derived feature reflecting the absence of standard ganglionic structuring in the mature nautiloid brain. Bullock [10], for example, has specifically proposed for the *Nautilus* brain that “a superficial resemblance to primitive medullary cords” may have been produced during development by a secondary spread of neurons into commissures and the process-rich cores of ganglia.

A second approach to the problem of cephalopod brain origins is to ask how the cephalopod brain develops. Is its central nervous system initially generated as a system of cords or as a spatially restricted set of nodal ganglia? Indeed, Bullock’s criticism of Young’s *Nautilus* analysis, that a commissural and longitudinal spread of neurons from paired ganglia could readily account for any cordal appearance of the adult cephalopod nervous system [7], is in essence a speculation about development.

Octopus and squid nervous system development has been studied using reconstructions of histological sections [11–16]. These reports concluded that coleoid brain development is ganglionic (Fig. 1*Right Upper Panel*), with neural progenitors arising in ectodermal thickenings and ingressing from the surface ectoderm. These ingressing cells form four pairs of clusters of neuronal cell bodies that initially lack processes. The anterior clusters, bilateral to the mouth, give rise to the cerebral ganglia, which later fuse across the midline. The other three ganglionic pairs become (1) the optic ganglia adjoining the embryonic eye field, (2) the pedal ganglia near the base of the arms, and (3) the palliovisceral or visceral ganglia situated along the collar between the mantle and the arms. In these histological reconstructions, the cerebral, pedal and palliovisceral clusters appear spherical or nodular in shape. Only the prospective optic ganglia display an elongated C-shape reminiscent of a medullary cord. The ganglia expand in size with the accumulation of additional neurons, eventually forming the masses of the adult cephalopod brain (Fig. 1*Right Lower Panel*).

Development of the coleoid brain from a system of cords would appear very different (Fig. 1*Middle Upper Panel*). The early allocation of neurons would be not to nodular clusters, but to extensive, rope-like territories in the neurectoderm. Some of these neuronal territories would be expected to span the midline early in embryogenesis, rather than populate the midline by ganglionic fusion later in development. The size of the cordal territories would increase over time, but adult brain morphology would arise from global transitions of the embryonic body plan that centralize the peripherally arranged cords, rather than relatively local and more constrained migrations of neuronal somata.

Because traditional silver staining methods for recognizing neurons do not work effectively on early cephalopod embryos, the reconstruction data supporting a ganglionic origin for the coleoid brain were based on material stained with alum-haematoxylin and toluidine blue [11–16]. Consequently, definitive cell-type identification of embryonic neurons was not possible, making the often-repeated conclusion of ganglionic rather than cordal origins of the coleoid brain less secure. In this report, we have revisited the issue of coleoid brain development by studying the expression of molecular markers of neuronal cell types in wholemount preparations of octopus embryos, which permits direct demonstration, rather than section reconstruction, of three-dimensional patterns.

*elav* (embryonic lethal, abnormal visual system) is a RNA binding protein gene identified in a *Drosophila* mutant screen as required by neurons from their birth [17]. Molecular analysis has demonstrated that *Drosophila* ELAV protein is expressed by young neurons, but not neuronal progenitors [18]. Independent studies in mammals identified the genes for the autoimmune antigens HuC and HuD as *elav* homologs that are also expressed in postmitotic neurons (*ELAVL3* and *ELAVL4*; [19–21]). It is now clear that *ELAV* exhibits highly conserved neuron-specific expression during development across animals as disparate as flies, worms, and vertebrates [22, 23]. Nomaksteinsky et al. [24] took specific advantage of the restricted neuronal expression of *elav* to demonstrate that the nervous system of adult acorn worms is centralized into dorsal and ventral cords.

NEUROD and NEUROGENIN (NEUROG) are members of the atonal basic helix-loop-helix transcription factor family involved in the neuronal differentiation and cell-type specification of the developing vertebrate nervous system [25]. In *Xenopus* and mouse, Neurogenin directly activates the transcriptional targets *NeuroD1* and *NeuroD4* to induce regulators of neuronal signaling, differentiation, and migration [26]. Both *NEUROD* and *NEUROG* are highly conserved across many metazoan lineages including sponges and sea anemones [27]. In the annelid worm *Platynereis dumerilii*, for example, *Neurogenin* has been shown to be expressed principally in the apical-most layer of cells of the prospective ventral nerve cord, while annelid *elav* is abundant in more basal cells [28].

We describe here the pattern of neurogenesis in octopus embryos and assess the processes by which the octopus brain is assembled during embryogenesis. Our findings, based on *ELAV, NEUROD,* and *NEUROG* gene expression and immunolabeling using the neuronal territory marker Distalless-like (DLL), provide support for the conclusions that (1) the cephalopod brain develops from concentric cords, and (2) a system of nerve cords is a feature of molluscan neuroembryogenesis shared by octopuses, monoplacophorans, polyplacophorans and some early branching gastropod clades, such as those in Patellogastropoda and Vetigastropoda [29].

## Materials and methods

### Collection of animals

**Fig. 2:**
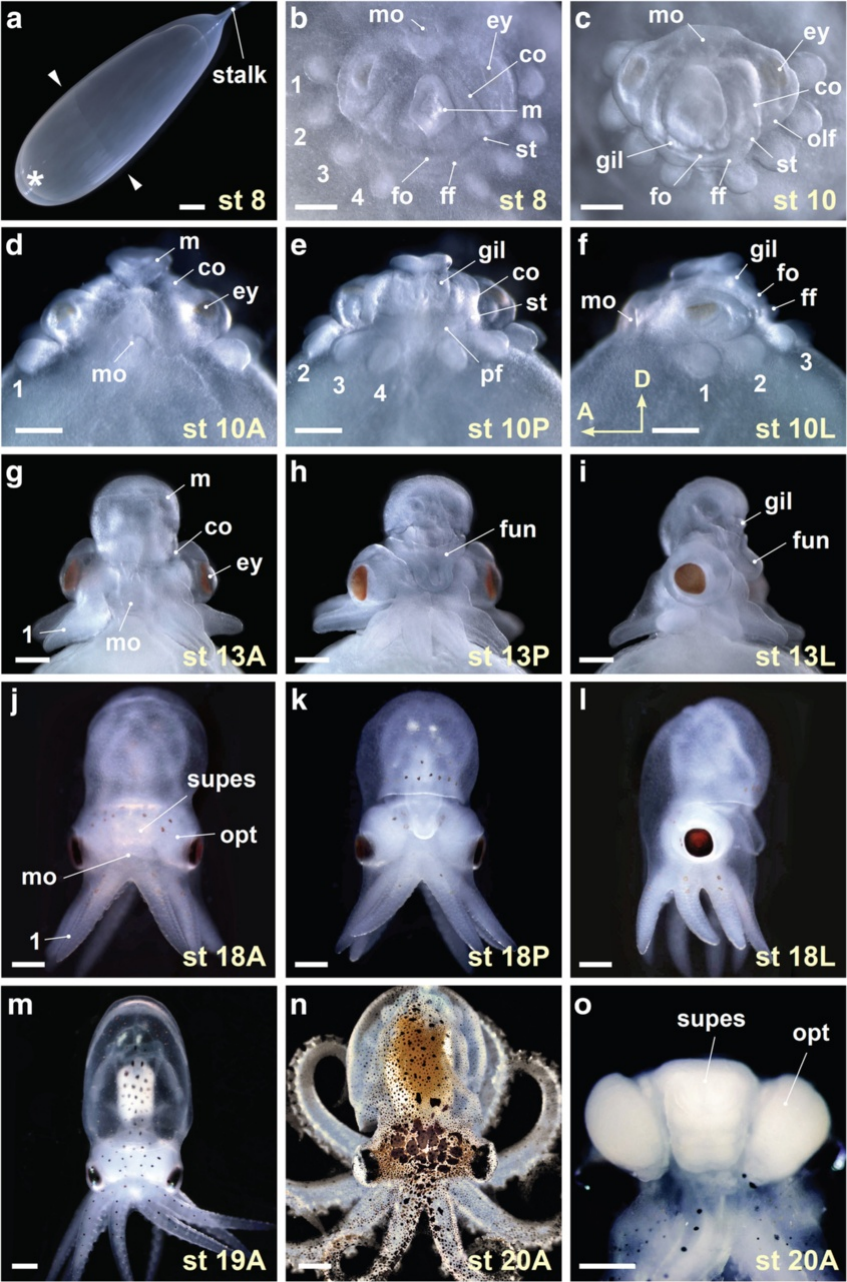
Developmental staging of *Octopus bimaculoides*. **a** Whole egg photomicrograph illustrates the egg stalk and the animal pole (asterisk) where the embryonic body forms. Extent of epiboly in this stage (st) 8 embryo is marked with arrowheads. **b** End on view of a stage 8 embryo with the egg capsule and yolk removed. In dark field illumination, the organ primordia are visible as ectodermal and mesodermal thickenings. The mantle anlage (m) is central, the prospective mouth (mo) at the top of the panel is anterior, and the arm bud pairs (1–4) are arrayed peripherally. The folds of the collar (co) and the prospective funnel (fo, ff) fall at intermediate positions. **c–f** The growth of the organ systems by stage 10 is illustrated in end on (**c**), anterior (**d**), posterior (**e**) and left side (**f**) views. **g–m** The shape of the adult octopus emerges at middle (**g–l**) and late (**m**) embryonic stages. Illustrated are anterior (**g** and **j**), posterior (**h** and **k**) and left side (**i** and **l**) views of stage 13 (**g–i**) and stage 18 (**j–l**) embryos, and an anterior view of a stage 19 embryo (**m**). **n** and **o** Anterior views of *O. bimaculoides* (**n**) and its brain (**o**) at hatching (stage 20). A, anterior view; ey, eye; fun, funnel; gil, gill; L, lateral view; olf, olfactory organ; opt, optic lobe; P, posterior view; pf, funnel pouch; st, statocyst; supes, supraesophageal mass. Scale bars: 1mm (**a**), 500 μm (**b–o**)

The Southern California two-spot octopus, *Octopus bimaculoides,* has been identified as a large-egg octopus species amenable to laboratory culture [30, 31]. The adult females spawn in intertidal and subtidal zones, and were obtained with their clutches from Aquatic Research Consultants (Dr. Chuck Winkler, San Pedro, CA). The transported adults and eggs were maintained at room temperature in a closed circular aquarium system filled with artificial seawater. To describe our findings, we followed the convention of embryological orientation [32] shown in Fig. 2, rather than the physiological orientation employed in most adult cephalopod scientific reports [33]. The anatomical terms follow Young [33] on adult octopus brain and Shigeno et al. [14] on the squid embryo. For the brain anlagen, however, we used the term ‘cord’ rather than ‘ganglion’ in accordance with our findings in *Nautilus* [34] and *Octopus* (this study).

### *ELAV* gene isolation

An 849bp PCR product, recovered from stage 18 octopus embryo cDNA, was identified as representing an octopus *ELAV* ortholog based on 71 % nucleotide and 68 % amino acid identity with the *ELAV* sequence from the annelid *Platynereis dumerilii* (GenBank EF384209). The PCR employed two rounds of nested primers. Primers were designed with Blockmaker and CODEHOP algorithms [35, 36] using *Drosophila*, mouse and *Amphioxus* amino acid sequences as inputs. First-round pairs were the forward primer 5′-GTGAACTACCTGCCCCARAMNATGAC-3′ (VNYLPQTMT) and the reverse primer 5′-GCGGCCTCGTCGTARTKNGYCAT-3′ (MTHYDEAA). Second-round nested primers were 5′-GGGCTACGGCTTCGTNAAYTAYRT-3′ (LGYGFVNY) and 5′-CACGGCGCCGAANGGNCCRAA-3′ (FGPFGAV). Primers were used at a final concentration of 3 μM. The PCR protocol for both rounds featured a 4-min denaturation step at 94 °C and 35 rounds of 35 s at 94 °C, 35 s at 42 °C, and 50 s at 72 °C. The amplified 849 bp band was excised from a 2 % low melt agarose gel and subcloned into the pCRII-TOPO vector (Invitrogen).

### Neuronal differentiation gene isolation

A *NEUROG* cDNA was recovered serendipitously during a quality control screen of 5′ RACE-ready *O. bimaculoides* cDNA synthesized with the SMART RACE kit (Clontech). An *O. bimaculoides NEUROD* fragment was amplified with the non-degenerate primers 5′-TTGGTGACAGCAATAGCG-3′ and 5′-GCGGATGATGACAGAATCTC-3′. *NEUROG* and *NEUROD* were subcloned into pCRII-TOPO (Invitrogen) and pGEM-T EASY (Promega), respectively.

### Phylogenetic analyses of gene families

Phylogenetic analyses of *NEUROD, NEUROG, ELAV,* and *ELAV-like* genes were performed to determine the orthology of the genes isolated. Related genes from other species were obtained from GenBank and aligned using MUSCLE [37]. Approximately maximum likelihood phylogenetic trees were generated with FastTree [38] and illustrated with FigTree (http://tree.bio.ed.ac.uk/software/figtree).

### Wholemount *in situ* hybridization

Embryos inside their egg cases were fixed by immersion in 4 % paraformaldehyde in phosphate-buffered saline (PBS) overnight at 4 °C. After removal of the chorions with fine forceps, the embryos were staged, sorted, and stored overnight at 4 °C in fresh fixative. Older animals (stage 20 embryos and hatchlings) were anesthetized in 3 % ethanol in seawater before fixation. Embryos and hatchlings were dehydrated through a methanol series prior to hybridization. Wholemount *in situ* hybridization was performed with digoxigenin-labeled riboprobes following previously established methods [39] with the following modifications: (1) embryos were bleached in 6 % hydrogen peroxide in PBS-1 % Tween (PBST) for one hour following rehydration, and (2) after detergent treatment young embryos were postfixed in 4 % paraformaldehyde-PBS without glutaraldehyde. Labeled RNA probes were detected with antibody-phosphatase conjugates (Roche Molecular Biochemicals, Indianapolis, IN), and enzyme-catalyzed deposition of chloride 5-bromo-4-chloro-3-indolyl phosphate (BCIP) and nitroblue tetrazolium (NBT). Control embryo wholemounts hybridized with *ELAV* sense probes did not display labeling above background (not illustrated).

### Combined immunostaining and gene expression histochemistry

Paraformaldehyde-fixed hatchlings were cryoprotected in 20 % sucrose-PBST, embedded in 10 % gelatin blocks, and cut into 30-micron thick sections on a freezing microtome. Sections were mounted on glass slides and processed for *in situ* hybridization [39]. After the tetrazolium histochemistry, the sections were rinsed in PBST, blocked in 10 % bovine serum-PBST and incubated for 3-6 hours at room temperature with the mouse monoclonal antibody 6-11B-1, which recognizes acetylated alpha-tubulin (1:5000 dilution in 1 % serum-PBST; Sigma, T6793). Antibody labeling was detected with goat anti-mouse IgG conjugated to Alexa 594 (Sigma, A11005).

### Wholemount immunostaining

Paraformaldehyde-fixed embryos (stage 8) were rinsed in PBST, blocked in 10 % bovine serum-PBST and incubated overnight at 4 °C with a rabbit polyclonal antibody against DISTALLESS (DLL) homeodomain [40] diluted 1:2000 (kindly provided by Dr. Claudia Farfán) and with the mouse monoclonal antibody for acetylated alpha-tubulin. Secondary antibody labeling for DLL employed a goat anti-rabbit IgG conjugated to Alexa 488 (Sigma, A11008). 40,6-diamidino-2-phenylindole dihydrochloride (DAPI, Sigma, 5 μg ml^−1^ in PBS) served as a fluorescent nuclear marker to illustrate the overall morphology of the embryo. Embryos were incubated in DAPI solution for 30 min, washed in PBS, and studied with UV stereo epifluorescence microscopy. Immunostained samples were analyzed with a TIRF Live Cell Microscope (Olympus) or Leica TCS SP2 AOBS laser scanning confocal microscope.

## Results and discussion

### Staging of *O. bimaculoides* development

The embryos of *O. bimaculoides* display the direct development mode typical of cephalopods, without the cataclysmic metamorphosis seen in other molluscan larvae [41, 42]. The placement of the early embryo is telolecithal, and the movements of gastrulation are epibolic. We adopted the morphological criteria defined for *Octopus vulgaris* [42] and *Eledone cirrosa* [43, 44] to stage *O. bimaculoides* development, recording Naef stages I–XX as 1-20 (Fig. 2). The embryo is first visible as an apical disc on top of the large yolk (Fig. 2a). As epiboly proceeds, concentrically placed organ primordia form, with the mantle at the center and the four pairs of arm buds at the periphery (Fig. 2b). In the intermediate region, the primordia of the collar and funnel are visible as distinct folds between the mantle and the arms (Fig. 2b, e, and h). Also visible in the intermediate region are the ectodermal thickenings that give rise to three major sensory organs: the eyes, the olfactory organs, and the statocysts (Fig. 2b and c). The morphogenetic movements that generate the adult octopus anatomy are extensive, but maintain the organ topology found in the organogenetic stage (stage 8) embryo. The mantle at the center of the disc extends upwards and the periphery of the disc contracts, establishing an arm crown around the mouth (Fig. 2d-n) and forming a circumesophageal brain comprising a supraesophageal mass with paired optic lobes (Fig. 2o) and a subesophageal mass with brachial, pedal, and palliovisceral lobes [33].

### *ELAV* mRNA labels somata but not processes in octopus brain

**Fig. 3:**
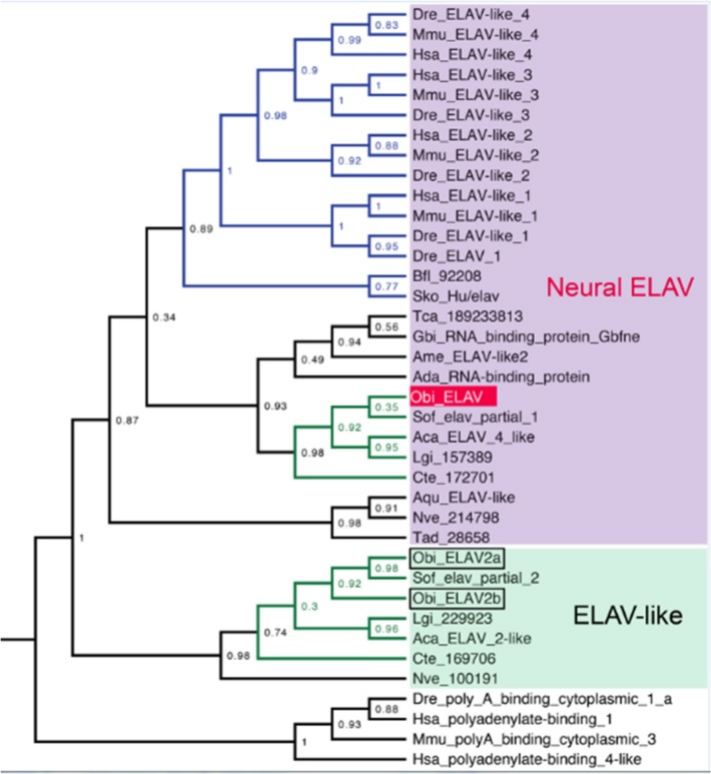
Phylogenetic analysis of *ELAV* and *ELAV-like* genes. Invertebrates, including sponges, cnidarians and placozoans, have a single neural *ELAV* gene, while jawed vertebrates have four or more. In addition, *ELAV-like* genes are present in cnidarians and lophotrochozoans, but not deuterostomes and (apparently) ecdysozoans. In *O. bimaculoides*, we find two *ELAV-like* genes and one neural *ELAV* gene. Relationships of metazoan *ELAV* and *ELAV-like* genes are illustrated by an approximately maximum likelihood tree with genes from *O. bimaculoides* (Obi), *Sepia officinalis* (Sof), *Lottia gigantea* (Lgi), *Aplysia californica* (Aca), *Capitella teleta* (Cte), *Saccoglossus kowalevskii* (Sko), *Branchiostoma floridae* (Bfl), *Danio rerio* (Dre), *Mus musculus* (Mmu), *Homo sapiens* (Hsa), *Tribolium castaneum* (Tca), *Gryllus bimaculatus* (Gbi), *Apis melifera* (Ame), *Anopheles darlingi* (Ada), *Amphimedon queenslandica* (Aqu), *Trichoplax adhaerens* (Tad) and *Nematostella vectensis* (Nve)*.* Likelihood support indicated at nodes

**Fig. 4:**
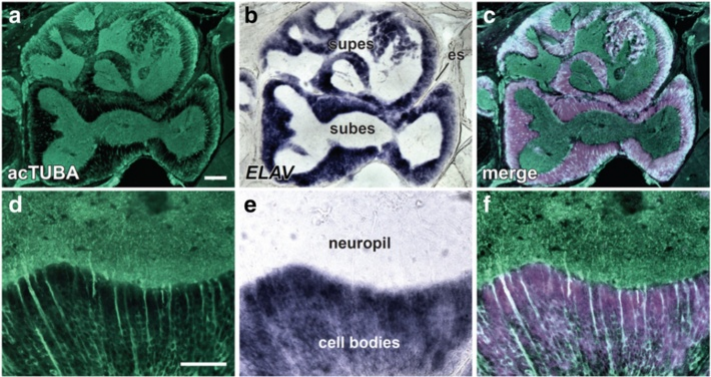
*ELAV* gene expression identifies neuronal somata in the brains of *O. bimaculoides* hatchlings. **a**–**c** Midline section taken through hatchling brain, with the mouth to the left. **d**–**f** High-power view of the transition between the rind of neuronal cell bodies and the neuropil core of the subesophageal mass. Section was double-stained for (**a** and **d**) anti-acetylated alpha-tubulin (acTUBA) and (**b** and **e**) *ELAV* gene expression. **c** and **f** *In silico* merging of fluorescence and light microscopic images. es, esophagus; subes, subesophageal mass; supes, supraesophageal mass. Scale bars: 100 μm (**a**) and 50 μm (**d**)

To study the early patterns of neuronal development, we isolated an *ELAV* cDNA fragment from *O. bimaculoides* (Fig. 3). We tested the specificity of *ELAV* gene expression for neuronal cell bodies in octopus hatchling brain. In *O. bimaculoides,* as in other coleoid cephalopods, the lobes of the mature brain display the typical architecture of invertebrate ganglia, with a rind of cell bodies surrounding a core of neuropil and fibers [4]. This arrangement was readily seen in hatchling brain sections immunostained for acetylated alpha-tubulin, which identifies neuronal processes in the lobular neuropil as well as neuronal cell bodies (Fig. 4a, d; see [33]). Section *in situ* hybridization experiments detecting *ELAV* transcripts demonstrated expression in all brain lobes examined (Fig. 4b). Single sections processed for acetylated alpha-tubulin immunohistochemistry and *ELAV* gene expression established that *ELAV* mRNA is a selective marker of octopus brain neuronal cell bodies and is not enriched in the processes of the lobular neuropil or in connectives (Fig. 4c, e and f; data not shown).

### *ELAV* gene expression in octopus brain development

**Fig. 5:**
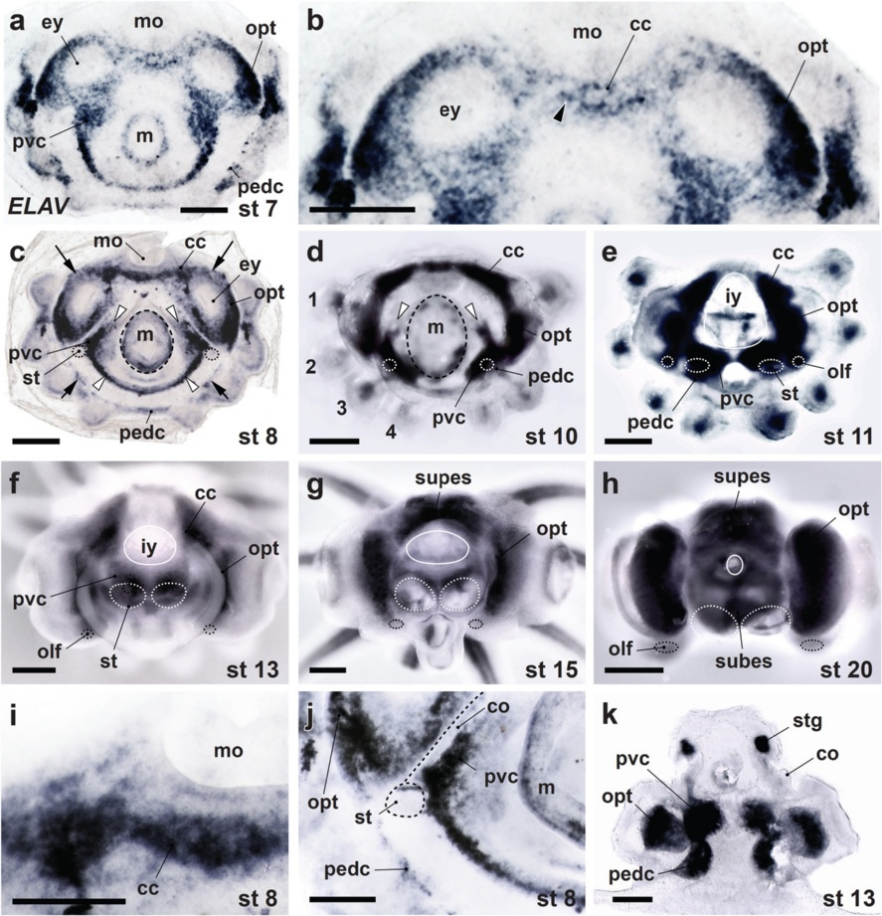
*ELAV* gene expression in *O. bimaculoides* embryos demonstrates the cordal development of octopus brain. **a–j** Wholemount *ELAV in situ* hybridization on early (**a–d, i** and **j**), middle (**e** and **f**) and late (**g**) stage embryos and hatchling brain (**h**). Orientation is an end on view as in Fig. 2b and c, with anterior to the top of the panel. **b, i**, and **j** High power images of cerebral cord (**b, i**) and palliovisceral cord (**j**) territories shown in **a** and **c** respectively. **k** Cross section taken through middle stage embryo wholemount, oriented with the mantle at the top as in Fig. 2g and h. Arrowhead in B shows *ELAV*-rich cells populating the midline at the earliest stage of detection. Arrows in C denote the lateral extent of the midline territories of early-born neurons in the cerebral cord (top, arrows with long stems) and the pedal cord (bottom, arrows with short stems). White arrowheads in C identify the limits of *ELAV*-positive cells extending from the concentrations of palliovisceral neurons inside the collar folds. White arrowheads in D point to the anterior limits of the palliovisceral cord. In **d**–**f**, the level of expression in the pedal cord is not faithfully documented because the pedal cord at these stages is in part shifted deep to the palliovisceral cord. The *ELAV* territories within the mantle identify the developing peripheral nervous system, including the prospective stellate ganglia (stg) illustrated in **k**. The limits of the mantle (m), statocysts (st) and olfactory organs (olf) are marked with dots. In **e**–**h**, the mantle has been cut away (solid circle, revealing the inner yolk, iy) so that the view of the brain primordia is unobstructed. 1-4, arms 1-4; cc, cerebral cord; co, collar; ey, eye; mo, mouth; opt, optic lobes; pedc, pedal cord; pvc, palliovisceral cord; subes, subesophageal mass; supes, supraesophageal mass. Scale bars: 500 μm (**a**–**h**, **k**), 250 μm (**i**, **j**)

To track the distribution of embryonic octopus brain neurons, we performed wholemount *in situ* hybridization analyses for *ELAV* expression in octopus embryos from stage 7 onwards (Fig. 5). *ELAV*-positive cells were widely distributed across the embryo when first detected at stage 7, and were not organized into bilateral pairs of cerebral, palliovisceral, or pedal ganglia, as predicted by the ganglionic origin hypothesis (Fig. 1*Right Upper Panel*). Instead, there were anterior and posterior bands of *ELAV*-rich cells spanning the midline (Fig. 5a, c and i), bilateral sets of multiple *ELAV*-rich cell clusters medial to the statocyst (Fig. 5c and j) and two curving bands of *ELAV* expression at the periphery of the embryonic disc near the arms (prospective optic lobes, Fig. 5a). The overall regional distribution of the *ELAV*-positive cells was broadly similar to that of brain precursors proposed in cytological studies in *Octopus vulgaris* by Marquis [13], providing further evidence that octopus *ELAV* expression is a reliable marker of young octopus neurons.

**Fig. 6:**
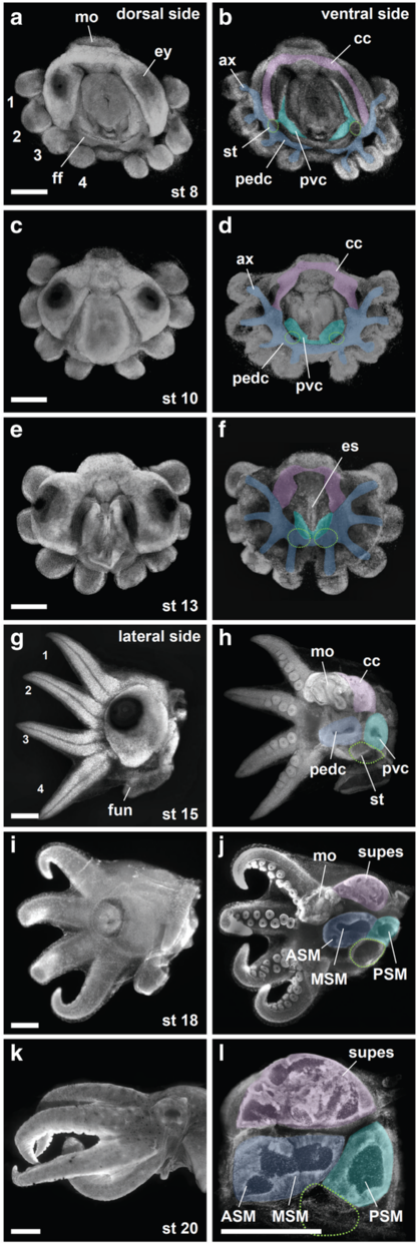
Compacting of the octopus brain cords. Brain development in the octopus is summarized with DAPI nuclear labeling imaged using UV stereo epifluorescence microscopy and pseudo-colored to indicate the locations of brain primordia. Pseudo-coloring is based on *ELAV* gene expression patterns and DLL immunocytochemistry studied in multiple embryo specimens from stage 8 to stage 20 (just before hatching). **a–f** Dorsal (left column) and ventral (right column) views of embryos from stage 8, 10 and 13. **g–l** Lateral views of embryos photographed from the side (left column) and after mid-sagittal cuts (right column). Outer yolk sacs were removed from the preparations for clarity. In L, only the central brain is shown. ASM, anterior subesophageal mass; ax, axial nerve cord; es, esophagus; MSM, middle subesophageal mass; PSM, posterior subesophageal mass. Other abbreviations as in Figs. 2 and 5. Scale bars: 500 μm (**a–l**)

By stage 10 (Fig. 5d, see also Summary Fig. 6), *ELAV*-positive cells were clearly distributed in a system of cords: an anterior cerebral cord and posterior palliovisceral and pedal cords, with the palliovisceral cord nested between the mantle and the pedal cord (compare Fig. 5d with Fig. 1*Middle Upper Panel*). *ELAV*-rich cell condensates were also visible in the arm buds. Comparison of *ELAV* expression patterns between stages 8 and 10 (Fig. 5c and d) indicated that, although neurogenesis is less advanced in the pedal cord and the arm buds than in other primordia, there are otherwise no pronounced global anterior-posterior or central-peripheral neurogenesis gradients. At stage 8, however, there were zones within each prospective cord that were enriched in *ELAV* gene expression, indicating the presence of within-cord neurogenetic gradients.

#### The cerebral cord

The cerebral cord is the anlage of the supraesophageal brain, including the learning and memory centers (frontal–vertical lobes) and the higher motor centers (basal lobes). This cord remains anterior throughout development. Previous descriptions suggested that anterior neurons are initially organized as paired spherical cerebral ganglia situated lateral to the foregut [12–14]. *ELAV* gene expression wholemounts demonstrated instead a cord-like arrangement as early as stage 7, when a band of *ELAV*-rich cells spans the midline immediately posterior to the mouth (Fig. 5a, b and i). Sites of early neurogenesis were also seen at the lateral limits of the prospective optic lobes, where *ELAV* gene expression identified a bilateral pair of swoosh-like territories. Between these three concentrations of early neurons, the densities of labeled cells appeared to decline gradually. By stage 10 the cerebral and optic lobe precursor territories extended circumferentially to form a nearly continuous cord of young neurons (Fig. 5d). Between stages 11 and 15, cerebral cord tissue condensed to form a supraesophageal mass flanked by the optic lobes (Fig. 5e–g; see also Summary Fig. 6).

#### The palliovisceral cord

The palliovisceral cord gives rise to the posterior subesophageal mass, which innervates all dorsal organs, including the mantle, visceral mass, and collar, through the pallial, visceral, and posterior funnel nerves [33]. *ELAV* gene expression at stages 7 and 8 demonstrated concentrations of prospective palliovisceral neurons beneath the presumptive mucous funnel organ at the posterior midline and in a bilateral pair of multiple cell-clusters just inside the collar folds (Fig. 5a, c, and j). In addition, low-density fields of *ELAV*-positive cells extended away from these concentrations in a graded fashion. By stage 10, *ELAV* expression clearly described a U-shaped palliovisceral cord encircling the prospective mantle, which was capped by the C-shaped cerebral cord (Fig. 5d). This neural architecture is strikingly different from the spherical ganglionic forms described for the developing palliovisceral nervous systems of *Octopus vulgaris* and the oegopsid squid *Todarodes pacificus* [13, 16], but is in closer accord with the report of an elongated palliovisceral primordium in the pygmy squid *Idiosepius paradoxus* [15]. At later stages, the palliovisceral cord condenses to form a pair of ovoid masses, which then centralize to produce a single posterior subesophageal lobe (Fig. 5e–h).

#### The pedal cord

This cord, also sometimes called the brachiopedal cord, forms a large brain center for the arms and anterior part of the funnel. The pedal cord is initially situated close to the arm buds and the prospective axial nerve cord of each arm, as previously reported [13]. By stage 8, *ELAV* gene expression demonstrated neurons extending from the prospective axial nerve cords to the pedal cord (Fig. 5c). *ELAV* labeling established that the pedal cord is continuous across the posterior midline, extending under the funnel folds (Fig. 5a, c). At later stages, the *ELAV*-rich pedal cord centralizes to form the compact structure comprising the anterior and middle subesophageal masses (see Summary Fig. 6).

### Markers of neurogenetic territories

**Fig. 7:**
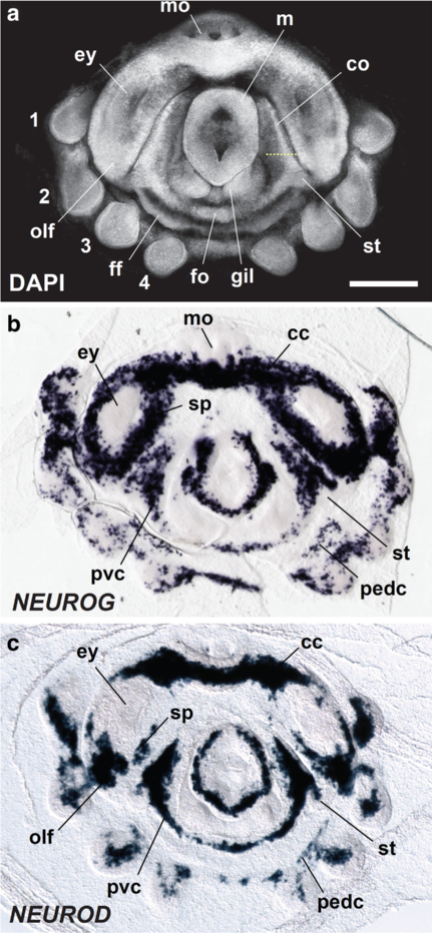
Neuronal differentiation gene expression also follows a cordal pattern. **a** DAPI nuclear labeling illustrates the external morphology of the embryo at stage 8. The dotted line indicates the positioning of the cross sections presented in Fig. 8. **b** and **c** Expression of *NEUROG* (**b**) and *NEUROD* (**c**) at stage 8 shown with wholemount *in situ* hybridization. All pictures are dorsal views and the mouth is at the top. sp, subpedunculate tissue (juxtaganglionic tissue). See abbreviations as in Figs. 2 and 5. Scale bar: 500 μm (**a**)

*ELAV* is an early marker of postmitotic neurons in vertebrates [20]. We investigated at embryonic stage 8 the spatial patterns of neurogenesis using DAPI nuclear staining and *in situ* hybridization for the neuronal determination genes *NEUROD* and *NEUROG* (Fig. 7a-c). Expression of *NEUROG* was strong in the prospective cerebral, palliovisceral, and pedal cords (Fig. 7b). *NEUROG* mRNA appeared to identify the origins of peripheral neurons of the mantle and arm buds as well as those of the olfactory organs, which are situated posterior to the eye placodes. A cordal expression pattern was also observed with *NEUROD* gene expression (Fig. 7c). To our knowledge, this is the first report of the expression of *NEUROG* and *NEUROD* genes in a mollusc [45].

**Fig. 8:**
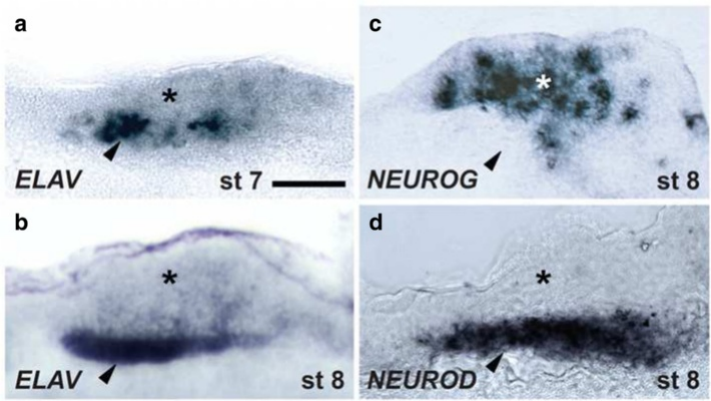
Apical-basal organization of neuroectoderm. **a** and **b** *ELAV*-expressing cells are found deep in the ectoderm from the time of earliest detection (**a**: stage 7; **b**: stage 8). **c** and **d** Expression patterns of *NEUROG* (**c**) and *NEUROD* (**d**) at stage 8. Cross sections illustrated are taken from the palliovisceral cord (see dotted line in Fig. 7a). The asterisks and arrowheads indicate putative neurogenic ectoderm apically and the basal site of neuronal differentiation, respectively. Scale bar: 20 μm

In tissue cross-sections (Fig. 8), we found that the developing cords exhibit apical-basal layering, one consistent with an apical proneural role in the ectoderm for *NEUROG* and with *ELAV*- and *NEUROD*-rich neurons migrating inwards to a basal position. This pattern is nearly identical to that reported in the polychaete *Platynereis dumerilii* and in the vertebrate neural tube [27, 28], suggesting that this apical-to-basal mode of molecular neurogenesis is shared by molluscs [45].

**Fig. 9:**
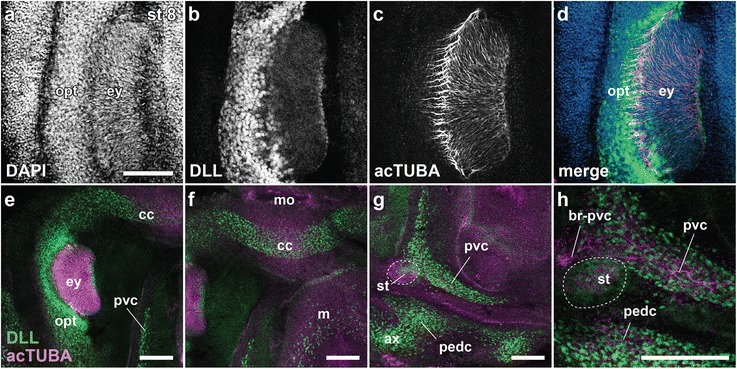
Immunohistochemistry for the early neural marker DLL provides further support for a cordal organization of octopus brain at stage 8. **a** DAPI nuclear labeling of a left eye demonstrates neural, retinal, and non-neural masses. **b–d** Confocal optical sections using anti-DLL (**b**, green in **d**), anti-acetylated alpha tubulin (**c**, acTUBA, purple in **d**) antibody staining, and the merged view (**d**) demonstrate the boundaries and distributions of the brain primordia and developing neural processes. **e–h** DLL and acTUBA double staining mark the cerebral (**e** and **f**), palliovisceral (**e**, **g** and **h**) and pedal (**g** and **h**) cords. Some neuronal fibers are seen in the enlargement view for the palliovisceral and pedal cords as indicated in H. br-pvc, brachio-palliovisceral connectives; other abbreviations as in Figs. 2 and 5. Scale bars: 100 μm

DLL immunoreactivity has been reported as a widespread marker of neurogenic territories in protostomes [40]. In octopus embryos, DLL immunostaining provided further support for a cordal pattern of neural development. In particular, DLL-rich cells filled the cerebral, optic, pedal, and palliovisceral territories at embryonic stage 8 (Fig. 9). From combined analyses of DLL immunohistochemistry with acetylated alpha-tubulin immunostaining for fibers, and DAPI labeling to mark transitions in cell density, a clear cord-like arrangement of the stage 8 embryo emerges (Fig. 9).

Acetylated alpha-tubulin-positive processes are prominent in the palliovisceral territories, in the pedal cord close to the statocysts, and in the optic lobes and retina, but not in the cerebral cord (Fig. 9e–h), raising the possibility of an asynchronous maturation of neuronal cells and processes, reminiscent of that described for neurogenesis in the cuttlefish *Sepia officinalis* [46, 47]*.* In the Buresi et al. report [47], the expression of *Sof-elav1* in *S. officinalis* embryos showed an asynchronous maturation of neurons across different brain centers. The *Sof-elav1* expression appeared first in the palliovisceral centers, and only subsequently in the cerebral and optic lobe territories [47]. Our findings on octopus acetylated alpha-tubulin immunoreactivity do indicate asynchrony in neuronal development between the cerebral and pedal cords. However, in contrast to the cuttlefish study, we identified a coincident onset of *ELAV* expression for the cerebral and palliovisceral cords at stage 7, suggesting that the timing of neurogenesis is different in these species, with postmitotic events in the octopus proceeding rapidly during stage 8.

## Conclusions

Our study of neurogenetic gene expression in octopus embryo wholemounts indicates that the anlagen of the octopus brain follow a multi-cordal plan rather than one of multiple spherical ganglia such as those seen in the adult nervous system architecture of gastropod species, such as *Aplysia*, *Lymnaea*, and *Helix* [48]. This finding, together with the cord-like organization postulated recently for *Nautilus* embryos [34], strongly suggests that a multiple cord origin for the cephalopod brain is a shared feature of extant cephalopods. Recent molecular phylogenetic studies establish a close relationship between cephalopods and monoplacophorans [29, 49, 50] and ratify fossil evidence that cephalopods evolved from a monoplacophoran-like mollusc [6, 51]. The cord-like organization of the embryonic cephalopod nervous system can therefore be directly compared with the cerebral, palliovisceral (or pleurovisceral) and pedal medullary cords described in embryonic and adult monoplacophoran and polyplacophoran nervous systems [52–59]. One compelling interpretation of these data is that cephalopods, despite the large size and compact organization of their brains, have retained an ancestral molluscan nervous system plan of multiple neural cords [55, 57, 59], and that this plan is particularly conspicuous in the embryo.

### Accession numbers

*Octopus bimaculoides ELAV, NEUROD* and *NEUROG* sequences are deposited as GenBank FJ861207, KR153584 and HM369392.

## Footnotes

1 We employ the terms “cords” and “ganglia” as defined by Richter et al. [5]. Neural structures are cords if they are organized as longitudinally and transversely distributed neuronal somata; ganglia are tightly packed, clearly demarcated clusters of neuronal somata with centrally placed neuropils. The central nervous system of chitons, for example, is considered to be composed of a primitive type of neural cord, lacking true ganglia [8], as illustrated in Fig. 1*Left Lower Panel*.

## References

[1] Wells MJ (1962). Brain and Behaviour in Cephalopods.

[2] Hanlon RT, Messenger JB (1996). Cephalopod Behavior.

[3] Hochner B, Shomrat T, Fiorito G (2006). The octopus: a model for a comparative analysis of the evolution of learning and memory mechanisms. Biol Bull..

[4] Nixon M, Young JZ (2003). The Brains and Lives of Cephalopods.

[5] Richter S, Loesel R, Purschke G, Schmidt-Rhaesa A, Scholtz A, Stach T, et al. Invertebrate neurophylogeny: suggested terms and definitions for a neuroanatomical glossary. Front Zool. 2010;7:29. 10.1186/1742-9994-7-29 PMC2996375 21062451

[6] Kröger B, Vinther J, Fuchs D. Cephalopod origin and evolution: A congruent picture emerging from fossils, development and molecules. Bioessays. 2011;33:602–13. 10.1002/bies.201100001 21681989

[7] Young JZ (1965). The central nervous system of *Nautilus*. Philos Trans R Soc Lond B Biol Sci..

[8] Faller S, Holger Roth B, Todt C, Schmidt-Rhaesa A, Loesel R (2012). Comparative neuroanatomy of Caudofoveata, Solenogastres, Polyplacophora, and Scaphopoda (Mollusca) and its phylogenetic implications. Zoomorphology.

[9] Sigwart JD, Sumner-Rooney LH, Schwabe E, Heß M, Brennan GP, Schrödl M (2014). A new sensory organ in “primitive” molluscs (Polyplacophora: Lepidopleurida), and its context in the nervous system of chitons. Front Zool..

[10] Bullock TH, Bullock TH, Horridge GA (1965). Mollusca: cephalopoda. Structure and Function in the Nervous Systems of Invertebrates.

[11] Marthy HJ (1987). Ontogenesis of the nervous system in cephalopods.

[12] Meister G (1972). Organogenese von *Loligo vulgaris* Lam. Zool Jb Anat..

[13] Marquis F (1989). Die Embryonalentwicklung des Nervensysem von *Octopus vulgaris* Lam. (Cephalopoda, Octopoda), eine histologische Analyse. Verhandl Naturf Ges (Basel).

[14] Shigeno S, Tsuchiya K, Segawa S (2001). Embryonic and paralarval development of the central nervous system of the loliginid squid *Sepioteuthis lessoniana*. J Comp Neurol..

[15] Yamamoto M, Shimazaki Y, Shigeno S. Atlas of the embryonic brain in the pygmy squid, *Idiosepius paradoxus*. Zool Sci. 2003;20:163–79. 10.2108/zsj.20.163 12655180

[16] Shigeno S, Kidokoro H, Tsuchiya K, Segawa S, Yamamoto M (2001). Development of the brain in the oegopsid squid, *Todarodes pacificus*: an atlas up to the hatching stage. Zool Sci..

[17] Jimenez F, Campos-Ortega JA (1987). Genes in subdivision 1B of the *Drosophila melanogaster* X-chromosome and their influence on neural development. J Neurogene..

[18] Robinow S, White K (1991). Characterization and spatial distribution of the ELAV protein during *Drosophila melanogaster* development. J Neurobiol..

[19] King PH, Levine TD, Fremeau RT, Keene JD (1994). Mammalian homologs of *Drosophila* ELAV localized to a neuronal subset can bind in vitro to the 3′ UTR of mRNA encoding the Id transcriptional repressor. J Neurosci..

[20] Kim CH, Ueshima E, Muraoka O, Tanaka H, Yeo SY, Huh TL (1996). Zebrafish elav/HuC homologue as a very early neuronal marker. Neurosci Lett..

[21] Wakamatsu Y, Weston JA (1997). Sequential expression and role of Hu RNA-binding proteins during neurogenesis. Development..

[22] Denes AS, Jekely G, Steinmetz PR, Raible F, Snyman H, Prud’homme B (2007). Molecular architecture of annelid nerve cord supports common origin of nervous system centralization in Bilateria. Cell..

[23] Meyer NP, Seaver EC (2009). Neurogenesis in an annelid: characterization of brain neural precursors in the polychaete *Capitella* sp. I. Dev Biol..

[24] Nomaksteinsky M, Rottinger E, Dufour HD, Chettouh Z, Lowe CJ, Martindale MQ (2009). Centralization of the deuterostome nervous system predates chordates. Curr Biol..

[25] Paridaen JTML, Huttner WB (2014). Neurogenesis during development of the vertebrate central nervous system. EMBO Reports.

[26] Seo S, Lim JW, Yellajoshyula D, Chang LW, Kroll KL (2007). Neurogenin and NeuroD direct transcriptional targets and their regulatory enhancers. EMBO J..

[27] Simionato E, Ledent V, Richards G, Thomas-Chollier M, Kerner P, Coornaert D, et al. Origin and diversification of the basic helix-loop-helix gene family in metazoans: insights from comparative genomics. BMC Evol Biol. 2007;7:33. 10.1186/1471-2148-7-33 PMC1828162 17335570

[28] Simionato E, Kerner P, Dray N, Le Gouar M, Ledent V, Arendt D, et al. Atonal- and achaete-scute-related genes in the annelid *Platynereis dumerilii*: insights into the evolution of neural basic-Helix-Loop-Helix genes. BMC Evol Biol. 2008;8:170. 10.1186/1471-2148-8-170 PMC2435551 18541016

[29] Zapata F, Wilson NG G, Howison M, Andrade SCS, Jörger KM, Schrödl M, et al. Phylogenomic analyses of deep gastropod relationships reject Orthogastropoda. Proc R Soc B. 2014;281:20141739. 10.1098/rspb.2014.1739 PMC4211456 25232139

[30] Forsythe JW, Hanlon RT. Effect of temperature on laboratory growth, reproduction and life span of *Octopus bimaculoides*. Mar Biol. 1988;98:369-379.

[31] Hanlon RT, Forsythe JW (1985). Advances in the laboratory culture of octopuses for biomedical research. Lab Anim Sci..

[32] Fiorni P (1978). Morphogenese der Tiere. G 5-I: Cephalopoden.

[33] Young JZ. The Anatomy of the Nervous System of *Octopus vulgaris*. Oxford: Clarendon; 1971.

[34] Shigeno S, Sasaki T, Moritaki T, Kasugai T, Vecchione M, Agata K (2008). Evolution of the cephalopod head complex by assembly of multiple molluscan body parts: Evidence from *Nautilus* embryonic development. J Morphol..

[35] Rose TM, Schultz ER, Henikoff JG, Pietrokovski S, McCallum CM, Henikoff S (1998). Consensus-degenerate hybrid oligonucleotide primers for amplification of distantly related sequences. Nucleic Acids Res..

[36] Henikoff S, Henikoff JG, Alford WJ, Pietrokovski S (1995). Automated construction and graphical presentation of protein blocks from unaligned sequences. Gene..

[37] Edgar RC (2004). MUSCLE: a multiple sequence alignment method with reduced time and space complexity. BMC Bioinformatics.

[38] Price MN, Dehal PS, Arkin AP (2010). FastTree 2-approximately maximum-likelihood trees for large alignments. PloS One.

[39] Grove EA, Tole S, Limon J, Yip L, Ragsdale CW. The hem of the embryonic cerebral cortex is defined by the expression of multiple *Wnt* genes and is compromised in *Gli3*-deficient mice. Development. 1998;125:2315–25. 10.1242/dev.125.12.2315 9584130

[40] Panganiban G, Irvine SM, Lowe C, Roehl H, Corley LS, Sherbon B, et al. The origin and evolution of animal appendages. Proc Natl Acad Sci. 1997;94:5162–6. 10.1073/pnas.94.10.5162 PMC24649 9144208

[41] Boletzky S. Biology of early life stages in cephalopod molluscs. Advances in Mar Biol. 2003;44:143–203. 10.1016/s0065-2881(03)44003-0 12846042

[42] Naef A (1928). Die Cephalopoden (Embryologie).

[43] Fuchs E (1973). Organo- und Histogenese des Darmsystems, embryonale Blutbildung und Dotterabbau bei *Eledone cirrosa* Lam. (Cephalopoda, Octopoda). Zool Jb Anat.

[44] Mangold K, Boletzky S, Froesch D (1971). Reproductive biology and embryonic development of *Eledone cirrosa* (Cephalopoda: Octopoda). Mar Biol.

[45] Hartenstein V, Stollewerk A (2015). The evolution of early neurogenesis. Dev Cell..

[46] Baratte S, Bonnaud L (2009). Evidence of early nervous differentiation and early catecholaminergic sensory system during *Sepia officinalis* embryogenesis. J Comp Neurol..

[47] Buresi A, Canali E, Bonnaud L, Baratte S (2013). Delayed and asynchronous ganglionic maturation during cephalopod neurogenesis as evidenced by *Sof-elav1* expression in embryos of *Sepia officinalis* (Mollusca, Cephalopoda). J Comp Neurol..

[48] Chase RB (2002). Behavior and its Neural Control in Gastropod Molluscs.

[49] Kocot KM, Cannon JT, Todt C, Citarella MR, Kohn AB, Meyer A (2011). Phylogenomics reveals deep molluscan relationships. Nature..

[50] Smith SA, Wilson NG, Goetz FE, Feehery C, Andrade SC, Rouse GW (2011). Resolving the evolutionary relationships of molluscs with phylogenomic tools. Nature..

[51] Yochelson EL, Flower RH, Webers GF (1973). The bearing of the new late Cambrian monoplacophoran *Knightconus* upon the origin of the Cephalopoda. Lethaia..

[52] Haszprunar G, Ruthensteiner B (2013). Monoplacophora (Tryblidia)—Some Unanswered Questions. Am Malacol Bull..

[53] Plate LH (1898). Die Anatomie und Phylogenie der Chitonen. Zool Jahrb.

[54] Voronezhskaya EE, Tyurin SA, Nezlin LP (2002). Neuronal development in larval chiton *Ischnochiton hakodadensis* (Mollusca: Polyplacophora). J Comp Neurol..

[55] Eernisse DJ, Reynolds PD, Harrison FW, Kohn AJ (1994). Polyplacophora. Microscopic Anatomy of Invertebrates, Mollusca I.

[56] Friedrich S, Wanninger A, Bruckner M, Haszprunar G (2002). Neurogenesis in the mossy chiton, *Mopalia muscosa* (Gould) (Polyplacophora): evidence against molluscan metamerism. J Morphol..

[57] Gillette R, Ladd PC (1991). The molluscan nervous system. Neural and Integrative Animal Physiology.

[58] Page LR (1993). Developmental analysis reveals labial and subradular ganglia and the primary framework of the nervous system in nudibranch gastropods. J Neurobiol..

[59] von Salvini-Plawen L. Zur Morphologie und Phylogenie der Mollusken: die Beziehungen der Caudofoveata und der Solenogastres als Aculifera, als Mollusca und als Spiralia. Z Wiss Zool. 1972;184:205–394.

